# *Thermomyces lanuginosus* lipase-catalyzed synthesis of natural flavor esters in a continuous flow microreactor

**DOI:** 10.1007/s13205-015-0355-9

**Published:** 2016-01-11

**Authors:** Ahmad Mohammed Gumel, M. S. M. Annuar

**Affiliations:** 1 Department of Biotechnology and Microbiology, Faculty of Science, Federal University, Dutse, 7156 Jigawa State Nigeria; 2 Faculty of Science, Institute of Biological Sciences, University of Malaya, 50603 Kuala Lumpur, Malaysia

**Keywords:** Lipases, Microreactor, Lecitase Ultra™, Esterification, Flavor esters, Biocatalysis

## Abstract

Enzymatic catalysis is considered to be among the most environmental friendly processes for the synthesis of fine chemicals. In this study, lipase from *Thermomyces lanuginosus* (Lecitase Ultra™) was used to catalyze the synthesis of flavor esters, i.e., methyl butanoate and methyl benzoate by esterification of the acids with methanol in a microfluidic system. Maximum reaction rates of 195 and 115 mM min^−1^ corresponding to catalytic efficiencies (*k*_cat_/*K*_M_) of 0.30 and 0.24 min^−1^ mM^−1^ as well as yield conversion of 54 and 41 % were observed in methyl butanoate and methyl benzoate synthesis, respectively. Catalytic turnover (*k*_cat_) was higher for methyl butanoate synthesis. Rate of synthesis and yield decreased with increasing flow rates. For both esters, increase in microfluidic flow rate resulted in increased advective transport over molecular diffusion and reaction rate, thus lower conversion. In microfluidic synthesis using *T. lanuginosus* lipase, the following reaction conditions were 40 °C, flow rate 0.1 mL min^−1^, and 123 U g^−1^ enzyme loading found to be the optimum operating limits. The work demonstrated the application of enzyme(s) in a microreactor system for the synthesis of industrially important esters.

## Introduction

Naturally occurring flavor esters are commonly used in pharmaceutical, food, fragrance, and cosmetics industries as aroma agents. Increasing industrial demand for flavor and fragrance esters is evidenced by their current global markets that stand at $21.8 billion (BBC Research 2012). This figure is projected to exceed $30 billion in 2017, with an estimated annual growth rate of 5.6 % from 2012 to 2017 (BBC Research 2012). Although these types of esters can be extracted from plant and animal sources, the procedure is not economically viable due to the minute quantities of the esters, and expensive and work-intensive process (Romero et al. 2005). On the other hand, the production of flavor esters via chemical synthesis route gives higher yield, but the process is not eco-friendly and the synthesized esters contain traces of toxic impurities, which may result in health complications (Cvjetko et al. 2012). This warrants the preferential use of biocatalytic process in the production of flavor esters.

Synthesis using microchannel reactor offers various advantages in the bioprocess design and production of fine chemicals. Among them include rapid and efficient mixing effects (Miyazaki and Maeda 2006), experimental procedure that could be precisely adjusted, high operating safety margin, and low risk of environmental pollution (Urban et al. 2006). Additionally, efficient mass and heat transfers provided by continuous flow microreactors as a result of large surface-to-volume ratio with regular flow profiles lead to improved yields and increased selectivity (Wirth 2008). Furthermore, microreactors were reported to provide superb reaction control through low system inertia attributed to the microvolume processing of the reactants at any given time (Ahmed-Omer et al. 2007). This is also said to offer a solution of less waste disposal as compared to the conventional methods (Mason et al. 2007). Due to these and many other reasons, the properties of a microreactor system are continuously being exploited for process intensification and scale-up (Roberge et al. 2005). In fact, continuous flow microreaction technology is now a recognized and efficient approach within the present biocatalytic research community (Wirth 2008).

Lipases (EC 3.1.1.3) from different organisms were reported to catalyze the synthesis of different industrially important chemicals and their intermediates (Han et al. 2011; Gumel et al. 2013a, b, c, 2015; Gumel and Annuar 2014) including flavor esters such as ethyl caproate (Alvarez-Macarie and Baratti 2000), *cis*-3-hexenyl acetate (Jin et al. 2012), hexyl octanoate (Lopresto et al. 2014), geraniol and citronellol (Claon and Akoh 1993), butyl-hexanoate (Talon et al. 1996), butyl-acetate, and propyl-acetate esters (Mahapatra et al. 2009). Similarly, Cvjetko et al. (2012) reported the use of *Candida antarctica* lipase B to catalyze the synthesis of isoamyl acetate in packed bed microreactor (Mason et al. 2007). Despite their industrial significance as natural flavor esters, the syntheses of methyl butanoate and methyl benzoate (oil of niobe) via enzymatic catalysis were scarcely studied. While emerging as one of a more versatile hydrolases for the applications in industrial fields (De Maria et al. 2007), the utilization of lipase from *Thermomyces lanuginosus* (Lecitase Ultra™ also known as Lipase TL) as catalyst in esterification reaction is not fully explored. This is even more so in continuous flow microreactor system where its application has never been investigated. In this study, we reported the enzyme-mediated syntheses of methyl butanoate and methyl benzoate flavor esters in continuous flow microreactor using lipase TL. Characterization of the synthesized esters and kinetic behavior of their syntheses were also investigated.

## Materials and methods

### Materials

Lipase from *Thermomyces lanuginosus* (Lecitase Ultra™) with commercial activity of 10 LU mL^−1^ was purchased from Sigma Aldrich (Prod. No. L3295), and benzoic acid (242381) and NaCl crystal window (Z112100-1EA) were purchased from Sigma Aldrich, USA. Dichloromethane (107020), butyric acid (800457), and methanol (106012) were purchased from Merck Millipore, Germany. All materials used were of analytical grade. Vibrio viscometer SV-10 (A & D Company Ltd., Japan) was used to measure the fluidic dynamic viscosity.

### Methods

#### Enzyme activity

The catalytic (esterification) activity of lipase TL was assayed according to the reported literature (Gumel et al. 2013a, b, c), using a microchanneled reactor system equipped with micromixers (LTF-Mx and LTF-Vs; Little Things Factory, Germany). The enzyme (30 µL mL^−1^ of reaction mixture) was added to a vial containing 10 mL of 10 mM 4-nitrophenyl palmitate solution in dichloromethane under continuous mixing and drawn into a 10-mL borosilicate glass syringe. 10 mL of 10 mM ethanol was drawn into another glass syringe. The contents of these two syringes were simultaneously fed into the microreactor at a flow rate of 0.5 mL min^−1^ using automatic infusion pump. Aliquots (50 µL each) of the reaction mixture were withdrawn at intervals and quenched by mixing with 1 mL of 0.1 M NaOH in a quartz cuvette. The 4-nitrophenol liberated by the reaction was measured at 412 nm (UV–Vis spectrophotometer V-630; Jasco, Japan) against a blank of distilled water. The enzyme activity was calculated as the initial slope of the progress curve of 4-nitrophenol liberation versus time.

#### General reaction procedure

**Fig. 1:**
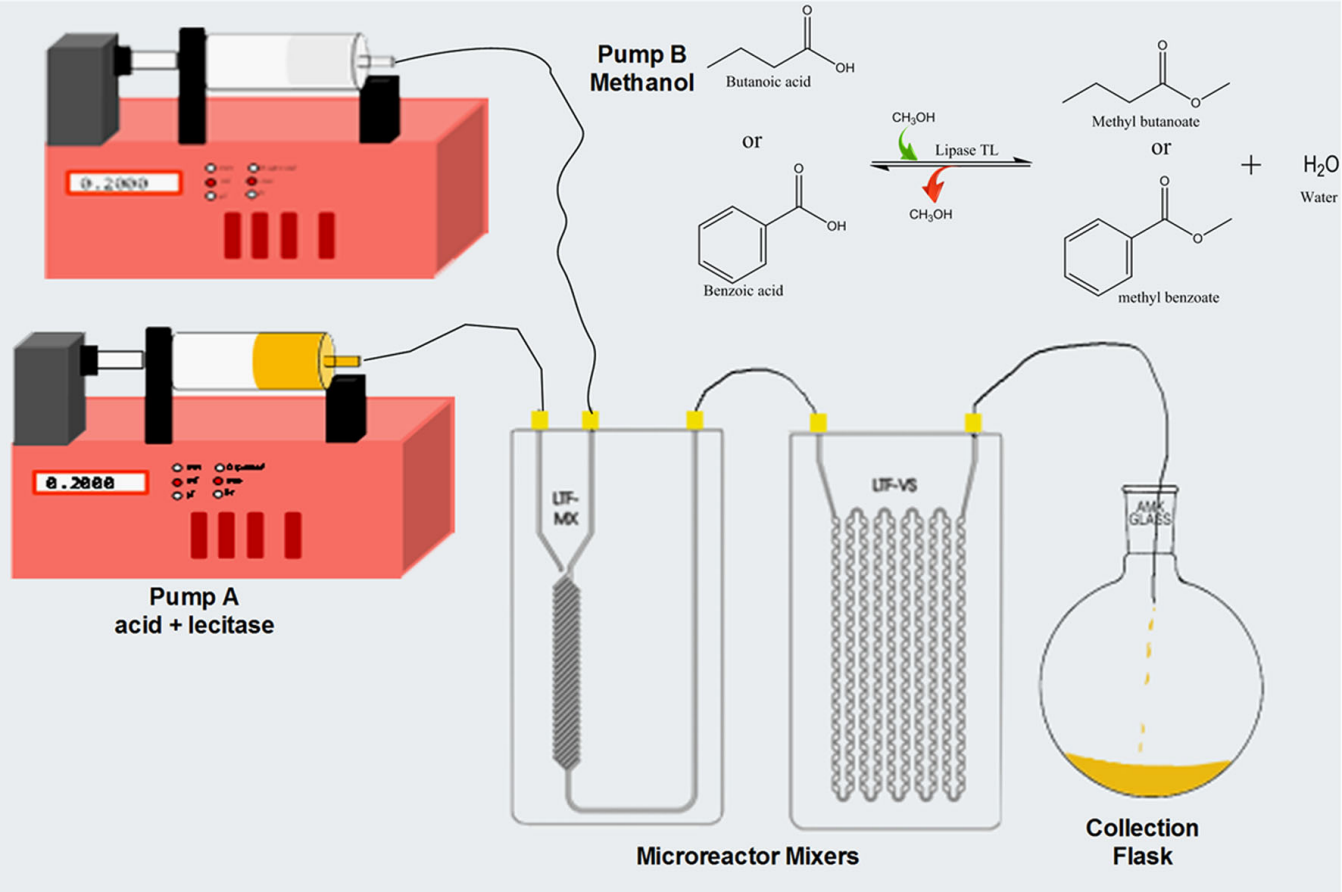
Schematic diagram of the microreactor setup

A specified concentration of either benzoic acid or butyric acid (7 mM) and 12 mM methanol was prepared in dichloromethane, each in a capped Scott bottles. Lipase TL (5 µL mL^−1^) was added under continuous magnetic stirring (200 rpm) to the bottle containing either butyric acid or benzoic acid. About 10 mL of each acid and alcohol solution were separately drawn into borosilicate glass syringe equipped with PTFE seal plunger (2624076; Duran, Germany) and mounted on an infusion™ automatic syringe pump (NE-300; New Era pump systems, USA) that was connected to a series of microchanneled reactor mixers (LTF-Mx and LTF-Vs; Little Things Factory, Germany) via PTFE microtubings (Ø ≈ 0.3 cm; Little Things Factory, Germany) as shown in Fig. 1. The reaction was carried out at 40 °C in a thermostat oil bath under continuous and simultaneous feeding of the reactants at a flow rate of 0.2 mL min^−1^. At regular interval, aliquot sample (100 µL) was taken at the outlet of the microreactor, diluted with 900 µL of dichloromethane and subjected to GC-MSMS analysis for residual fatty acid quantification. The fatty acid material balance was used to calculate the average volumetric rate of reaction (mM min^−1^). At the end of the reaction, the enzyme was centrifuged out of reaction mixture at 10,000×*g* for 10 min (Jiang et al. 2004). The crude esters were obtained by evaporating the supernatant containing the residual fatty acids and alcohol under reduced pressure and dried under vacuum. All reactions were carried out as described above unless stated otherwise.

#### Product characterization and analyses

Synthesized flavor esters were characterized using nondestructive FTIR-ATR performed on Perkin-Elmer Spectrum 400 FT-IR and FT-NIR Spectrometer (Perkin–Elmer Inc., Wellesley, MA, USA), equipped with PIKE GladiATR™ hovering monolithic diamond ATR accessory (Pike technologies Inc. USA). Briefly, aliquot sample (100 µL) of the crude extract was placed on the monolithic diamond ATR probe against the diamond crystal head. Thereafter, the samples were scanned at room temperature (25 °C) over a range of 4000–400 cm^−1^ as reported in the literature (Gumel et al. 2014).

Proton (^1^H) NMR analyses were recorded on JEOL JNM-GSX 270 FT-NMR (JOEL Ltd., Tokyo, Japan) machine at 250 MHz. About 1 µL of the crude ester was dissolved in deuterated chloroform (2 mL) containing tetramethylsilane (TMS) as the internal standard reference. The dissolved mixture was then filtered into an NMR tube using a glass syringe equipped with a 0.22 µm PTFE disposable filter (11807-25, Sartorius Stedim, Germany).

GC-MSMS analysis was carried out using Agilent triple quadrupole 7000B GCMSMS (Agilent, USA) equipped with GCMS triple axis detector carrying Agilent HP-5 ms capillary column (30 m long, 0.25 mm internal diameter, and 0.25 µm film thickness). The GC-MSMS injection and ramping method were conducted according to the previously reported literature (Gumel et al. 2013a, b, c).

#### Numerical calculations

As reported previously, the intrinsic kinetics of homogeneous catalysis (a reaction using free enzyme) is normally described using Michaelis–Menten model as shown in Eq. 1 (Segel 1993).

1 $$v = \frac{{V_{ \hbox{max} } \left[ S \right]}}{{K_{\text{M}} + \left[ S \right]}}$$

where *S* is the substrate concentration, *v* is enzyme reaction rate, *V*_max_ is maximum enzyme reaction rate, while *K*_M_ is the Michaelis–Menten constant, respectively.

In heterogeneous catalysis, the nature of the mass transport, diffusion limitation, and interactions between the reactants and the microfluidic channel is demonstrated using a series of correlations.

The mass diffusivity or diffusion coefficient (*D*) in cm^2^ s^−1^ was calculated using Lusis–Ratcliff model (Eq. 2) as described in the literature (Lusis and Ratcliff 1968).

2 $$D = \frac{{8.52 \times 10^{ - 8} \times T}}{{\eta_{\text{S}} (\bar{V}_{\text{S}} )^{1/3} }} \times \left[ {1.4 \times \left( {\frac{{\bar{V}_{\text{S}} }}{{\bar{V}_{\text{r}} }}} \right)^{1/3} + \frac{{\bar{V}_{\text{S}} }}{{\bar{V}_{\text{r}} }}} \right]$$

where $\bar{V}_{\text{S}} ,\;\bar{V}_{\text{r}}$ represent the molar volume (cm^3^ mol^−1^) of both solvent and reactant, respectively. The absolute temperature is denoted by *T* (K), and $\eta_{\text{S}}$ is the dynamic viscosity of solvent in centipoise (mPa s).

The effective diffusivity (*D*_eff_) described as the diffusivity within the streaming fluid was calculated according to Tabeling (2010) as shown in Eq. 3.

3 $$D_{\text{eff}} = D\left( {1 + \beta \left( {\frac{U}{D}} \right)^{2} } \right)$$

where *U* is the superficial velocity (cm s^−1^) and *β* is a coefficient that depends on the nature of the microfluidic canal as given by 0.02083 (Tabeling 2010).

The superficial velocity (*U*) was calculated according to Eq. (4) as described by Fogler (2011).

4 $$U = \frac{{q_{\text{v}} }}{A}$$

where *q*_v_ is the volumetric flow rate in cm^3^ s^−1^ and *A* is the cross-sectional area in cm^2^.

The calculated diffusion coefficient, *D*, was used in Eq. (5) to calculate the Damköhler numbers (*Da*). The *Da* parameter describes the influence of the fluidic transport phenomena in relation to the reaction rate.

5 $$Da = \frac{{D \times t_{\text{r}} }}{{l^{2} }}$$

where *t*_r_ is the characteristic reaction time (s) and *l* is the channel characteristic length (cm) calculated using Eq. 6. Thus, at *Da* ≪ 1, the diffusion rate signified to be higher than the reaction rate in the system, thus, the lower substrate conversion. Alternatively, at *Da* ≫ 1, the reaction rate is greater than the diffusion rate; thus, mixing and transporting in the system are said to be diffusion limited.

6 $$l = \frac{4r}{P}$$

where *r* and *P* are radius and channel circumference, respectively.

The *D* was then used to calculate the dimensionless Péclet number (*Pe*) as presented in Eq. 7.

7 $$Pe = \frac{U \times l}{D}$$

Thus, *Pe* ≫ 1 means the system is governed by advection rather than diffusion. On the other hand, if *Pe* ≪ 1, diffusion dominates transport over advection resulting in homogenous concentration.

The nature of the microfluidic flow was further ascertained using Reynolds number as illustrated in Eq. 8.

8 $$Re = \frac{U \times l}{{\nu_{\text{mixture}} }}$$

where, *ν*_mixture_ (Nu) is the kinematic viscosity of the mixture in cm^2^ s^−1^, which was calculated using Chevron Viscosity Blending Index (VBI) according to Maples (2000) models as illustrated in Eq. 9 through 11.

9 $${\text{VBI}}_{i} = \frac{{\ln \nu_{i} }}{{\ln (\nu_{i} \times 1000)}}$$

10 $${\text{VBN}}_{\text{mixture}} = \mathop \sum \limits_{i = 0}^{N} v_{i} \times {\text{VBI}}_{i}$$

11 $$\nu_{\text{mixture}} = \exp \left( {\exp \left( {\frac{{{\text{VBN}}_{\text{mixture}} - 10.975}}{14.534}} \right)} \right) - 0.8$$

where *v*_*i*_ is the individual components’ kinematic viscosity in centistokes.

## Results and discussion

### Product characterization

**Fig. 2:**
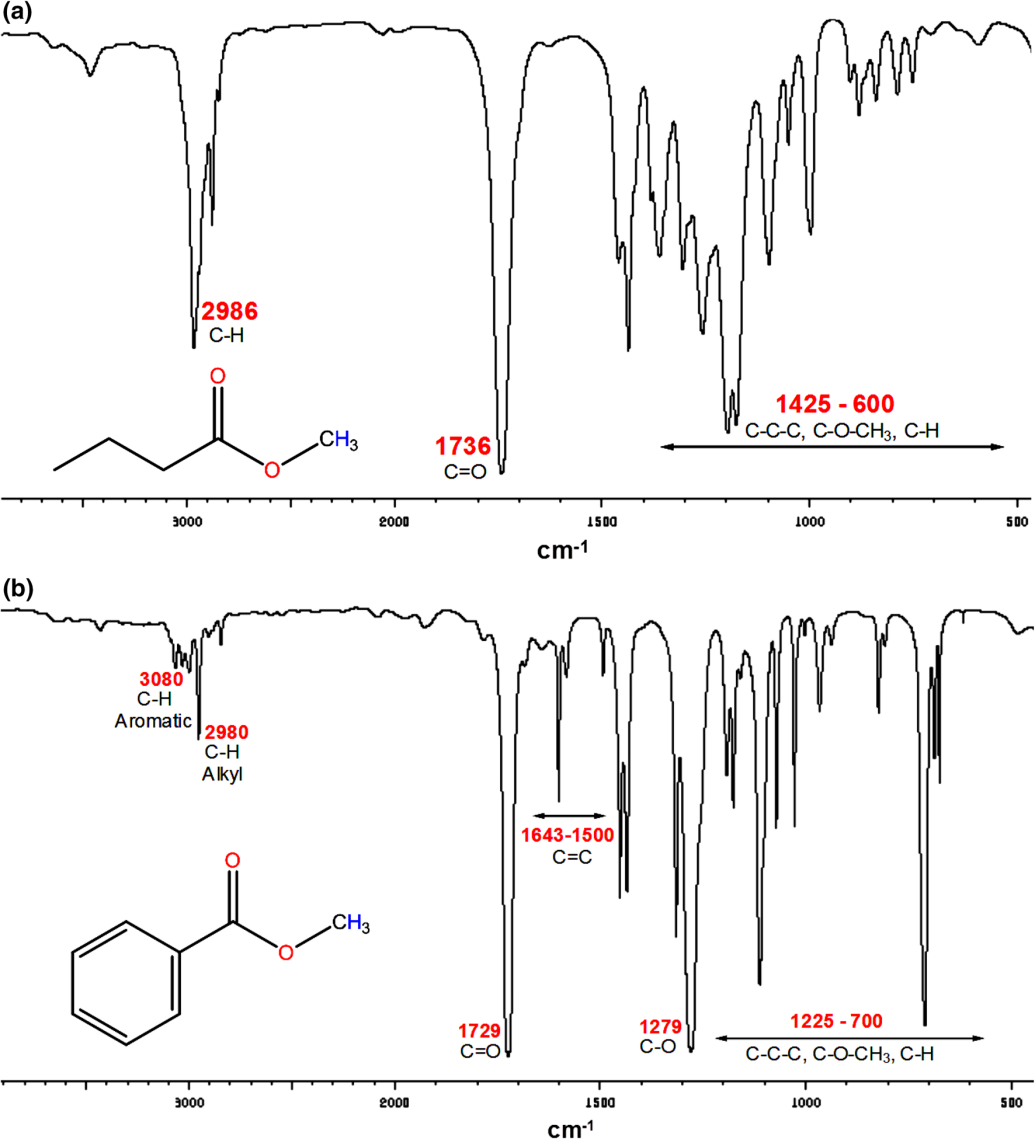
FTIR-ATR spectrum of **a** methyl butanoate and **b** methyl benzoate

In the FTIR spectra (Fig. 2a, b), the stretching vibration at 2686 and 2980 cm^−1^ wa assigned to alkyl C–H in both methyl butanoate and methyl benzoate, respectively. The stretching vibration at 3080 cm^−1^ was assigned to aromatic C–H in the methyl benzoate (Fig. 2b). The absorptions at 1736 and 1729 cm^−1^ were assigned to ester stretching vibration in both Fig. 2a and b, respectively. These assignments were found to be in agreement with the previously reported literatures (Sundaraganesan and Dominic 2007; Larkin 2011).

**Fig. 3:**
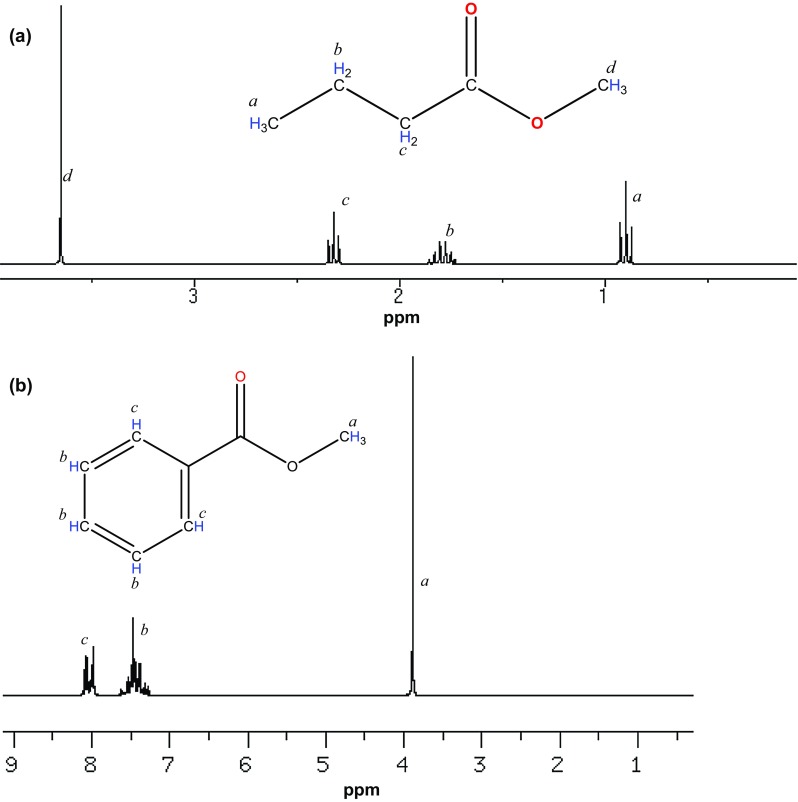
NMR spectrum of **a** methyl butanoate and **b** methyl benzoate

The synthesized flavor esters were further characterized using proton NMR analyses (Fig. 3a, b). In Fig. 3a, chemical shift “*a*” at 0.89 ppm is assigned to terminal beta methyl protons (–C–R) in methyl butanoate. Chemical shifts “*b*” and “*c*” were assigned to both (α, β) methylene protons [–C, –C(=O)O–C] of methyl butanoate (Fig. 3a). Chemical shifts designated by “*d*” at 3.67 ppm (Fig. 3a) and “*a*” at 3.89 ppm (Fig. 3b) were assigned to alpha ester-methyl protons [–OC(=O)C] in both methyl butanoate and methyl benzoate, respectively. Series of chemical shifts at 7.25–7.61 ppm designated by “*b*” were assigned to methine protons on carbon 3, 4, 5 of the annulene ring. Finally, chemical shift “*c*” at 8.22 ppm is assigned to annular methine protons adjacent to carboxylate on carbon 2 and 6 (Fig. 3b).

The spectroscopic data also indicated the absence of unwanted by-product(s) due to side reaction(s), a problem that is frequently encountered in chemical synthesis.

### Reaction kinetics

For the sake of efficient process development and optimization, it is interesting to observe via kinetic model the interaction between the freely diffused enzyme (i.e., lipase TL) and the reaction species as well as their transport phenomena and localization within the microfluidic channel of the reactor. The kinetic data were fitted to models based on freely diffuse Michaelis–Menten model. The mass diffusivity (*D*) of the reactant was empirically calculated using Lusis–Ratcliff correlation. Using the obtained diffusivity coefficient, Péclet and Damköhler numbers were determined. These numbers provide an insight on the reactants transport phenomena and catalytic behavior of lipase TL within the microflow setup. Table 1 presented a comparison of kinetics parameters observed during the ester synthesis. In this study, the enzymatic esterification catalytic efficiency, *E*_cat_ (min^−1^ mM^−1^), and the turnover number, *k*_cat_ (min^−1^), were also calculated according to the previous work (Gumel et al. 2013a, b, c).

**Table 1:** Comparison of kinetics and fluidic transport parameters between methyl butanoate and methyl benzoate esters (maximum standard deviation for kinetic parameters of M–M ≪ 1 %)

Kinetics parameters	Methyl butanoate		Methyl benzoate	
	M–M model	*R* ^2^	M–M model	*R* ^2^
*K*_M_ (mM)	203		150	
*V*_max_ (mM min^−1^)	195	0.995	115	0.988
*k*_cat_ (min^−1^)	60.2		35.5	
*E*_cat_ (min^−1^ mM^−1^)	0.30		0.24	
*Pe*	2.76 × 10^5^		3.07 × 10^5^	
*Da*	1.43 × 10^−3^		1.29 × 10^−3^	
*Re*	1.03 × 10^3^		1.03 × 10^3^	
*D* (cm^2^ s^−1^)	2.94 × 10^−5^		2.64 × 10^−5^	
*D*_eff_ (cm^2^ s^−1^)	4.59 × 10^5^		5.11 × 10^5^	

*M–M* Michaelis–Menten, *R* ^2^ coefficient of correlation

A glance at Table 1 suggests the Michaelis–Menten model adequately described the esterification reaction to produce both flavor esters (*R* ^2^ 0.988–0.995). Study of the kinetics parameters such as *K*_M_ (203 and 150 mM) and *E*_cat_ (0.30 and 0.24 min^−1^ mM^−1^) revealed the enzyme to have high substrate affinity and specificity. While the *K*_M_ values for different acyl donors used (butyric acid and benzoic acid) were comparable, the *V*_max_ value for esterification of methyl butanoate (195 mM min^−1^) was approximately twice of that methyl benzoate (115 mM min^−1^) (Table 1). This observation was attributed to the enumerated *k*_cat_ value of the enzyme for the substrates. The *k*_cat_ value of 60.2 min^−1^ was calculated during the synthesis of aliphatic methyl butanoate as compared to 35.5 min^−1^ in aromatic methyl benzoate (Table 1). The lower *k*_cat_ for methyl benzoic synthesis could be attributed to either the structural difficulty of benzoic acid to re-orientate itself within the active site or substrate specificity of the enzyme. This resulted in reduced chances and frequency of successful esterification with methanol compared with the aliphatic butyric acid. Concomitantly, this was further compounded by the observed higher *E*_cat_ in methyl butanoate synthesis (0.30 min^−1^ mM^−1^) in comparison with the observed *E*_cat_ of 0.24 min^−1^ mM^−1^ in methyl benzoate synthesis (Table 1). Based on the presented kinetics data, it could easily be perceived that the esterification reaction for both ester products showed freely diffuse, well-mixed enzyme behavior rather than apparent immobilized behavior.

However, despite the observed values of kinetics parameters, in this process, the kinetics data have to be use with caution to assess the system. For example, the enumerated Reynolds number (1030) indicates the fluidic transport within the microchannel is laminar in both processes. Yet, a closer look at the nature of dispersion transport of the processes revealed higher Péclet number (*Pe* ≫ 1) with corresponding low Damköhler number (*Da* ≪ 1). Thus, illustrating the process to be largely derived by advection mechanism, with higher rate of diffusion (Table 1). Interestingly, the empirically calculated molecular diffusivities (*D* and *D*_eff_) were found to agree with this observation. As it can be seen, the diffusion coefficient values (*D*) in both butanoic (2.94 × 10^−5^ cm^2^ s^−1^) and benzoic (2.64 × 10^−5^ cm^2^ s^−1^) acids were very low and in accord with the liquid diffusivity value reported in the literature (Tabeling 2010). As expected, the stream flow effective diffusivity (*D*_eff_) was found to be manifold higher than the calculated diffusion coefficient (Table 1).

### Influence of flow rate on reaction rate and substrate conversion

**Fig. 4:**
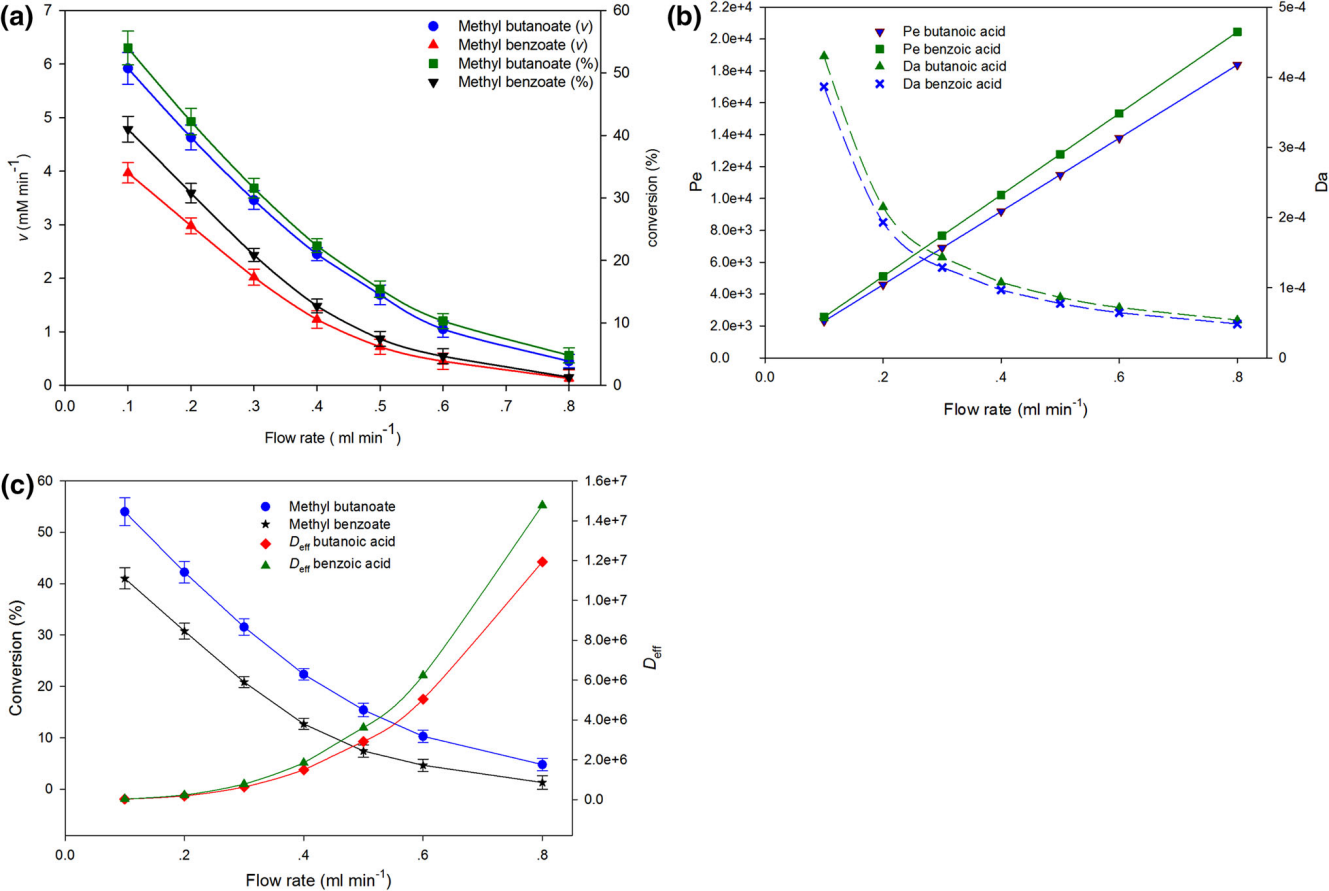
Fluid flow rate as a function of reaction rate and conversion. **b** Fluid flow rate as a function of Péclet (*Pe*) and Damköhler (*Da*) numbers. **c** Correlation between substrate conversion and effective diffusivity (*D*_eff_) as a function of flow rate. Reaction conditions: acid (7 mM), methanol (12 mM), 30 µL mL^−1^ lipase TL, and 40 °C

The influence of microfluidic flow rate on reaction and transport parameters was studied by varying the flow rate from 0.1 to 0.8 mL min^−1^ (Fig. 4). In lipase TL-catalyzed synthesis of methyl butanoate, operating at lower flow rate (0.1 mL min^−1^) resulted in an observed reaction rate of 5.9 ± 0.3 mM min^−1^ with corresponding conversion of about 54 %. This observation was found to be higher than the observed reaction rate of 3.9 ± 0.2 mM min^−1^ and conversion of 41 % for methyl benzoate synthesis under similar reaction conditions (Fig. 4a). For a given flow rate, both reactions were carried out under very similar fluid flow behavior. In a fixed microreactor channel length, increasing the flow rate shortens the residence time of the reactants, thus resulting in reduced rate and conversion yield. As expected, when the flow rate was increased from 0.1 to 0.8 mL min^−1^, a progressive decrease in both reaction rate and conversion was observed (Fig. 4a), culminating in lowest values of reaction rate (0.4 and 0.1 mM min^−1^) and conversion (4.8 and 1.3 %) in methyl ester of butanoate and benzoate syntheses, respectively. These observations were further substantiated by the observed change in transport phenomena with increasing flow rate (Fig. 4b, c). Increasing the flow rate from 0.1 to 0.8 mL min^−1^ resulted in a progressive increase in Péclet number with pronounced reduction in Damköhler numbers (Fig. 4b). Tabeling (2010) observed that the higher the Péclet number the higher the advective transport dominating the molecular diffusion. This was further found to be in accordance with the observed progressive reduction in Damköhler number (Fig. 4b) and increased effective diffusivity (Fig. 4c) at higher flow rate, illustrating the occurrence of diffusivity much faster than the reaction rate, thus the observed lower conversion.

Kataoka et al. (2009) reported similar trend in the catalytic activity of *Phycomyces nitens* lipase in mesoporous silica-packed microreactor. The study reported a steady decrease in reaction rate with increasing flow rate from <0.5 to 12 µL min^−1^. On evaluating the kinetics of horseradish peroxidase and β-glactosidase in microfluidic reactor, Seong et al. (2003) reported about 62 % reduction in the conversion of 0.5 µM H_2_O_2_ substrate when the flow rate was increased from 0.2 to 1.5 µL min^−1^. Similar observation on increased conversion yield with longer residence time was also reported in Novozym 435-catalyzed polymerization of ε-caprolactone in microreactor (Kundu et al. 2011).

### Effect of substrate molar ratio on esterification catalytic efficiency

**Fig. 5:**
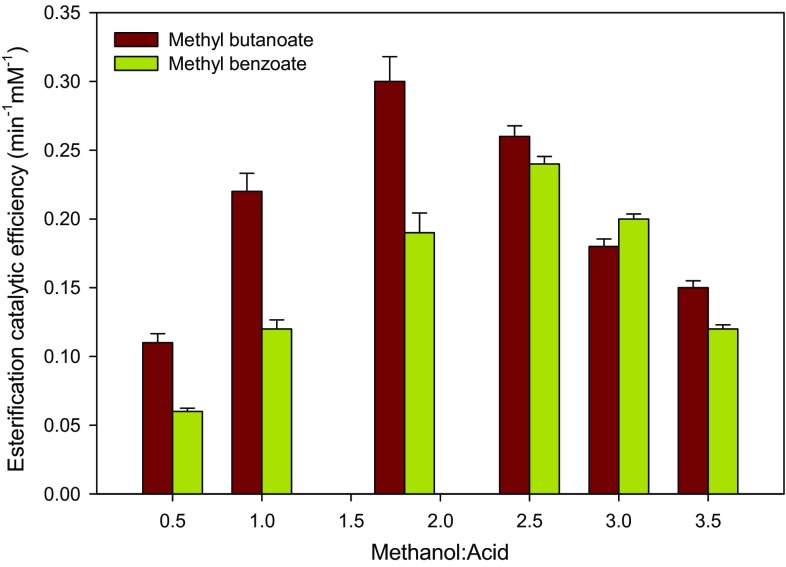
Substrate molar ratio as a function of esterification catalytic efficiency. Reaction conditions: 30 µL mL^−1^ lipase TL, 0.1 mL min^−1^ flow rate, and 40 °C

In enzymatic catalysis, the substrate relative amount is critical in determining the final product composition and the enzymatic esterification efficiency (Wang et al. 2011). The effect of substrate molar ratio on lipase TL catalytic efficiency in esterification was studied using different molar ratios of alcohol to acid at fixed acid concentration (Fig. 5). An increase in the catalytic efficiency of both esterifications was observed with increasing alcohol fraction up to a certain extent. In methyl butanoate synthesis, the catalytic efficiency increased with molar ratio up to 1.8 with maximum value of 0.30 min^−1^ mM^−1^. However, the esterification efficiency reduced markedly beyond 1.8 molar ratio with lowest value of 0.15 min^−1^ mM^−1^ at molar ratio of 3.5 (Fig. 5).

In contrast to methyl butanoate synthesis, the maximum esterification catalytic efficiency of 0.24 min^−1^ mM^−1^ was observed at molar ratio of 2.5 in methyl benzoate synthesis. Thereafter, the efficiency decreased gradually to a minimum value of 0.12 min^−1^ mM^−1^ at molar ratio of 3.5 (Fig. 5). Generally, the observed increase in esterification efficiency with increasing methanol ratio to a certain level could be attributed to the stability of some lipases in methanol to a certain limit. Santambrogio et al. (2013) have demonstrated that lipase from *Burkholderia glumae* is quite stable in the presence of methanol. In fact, the authors have shown that the lipase is capable of producing 90 % transesterification yield in the presence of about 75 % methanol loading. In contrast, the observed decrease in the esterification catalytic efficiency could be due to the fact that short chain alcohol deactivates lipase esterification activity by various mechanisms. High alcohol concentration was shown to lead to partial unfolding of enzymatic protein, which later leads to irreversible deactivation (Gumel et al. 2011a, b; Lotti et al. 2015). Studies using molecular dynamics simulations and experimental static light-scattering revealed that adsorption of alcohol molecules onto hydrophobic surfaces of the enzymatic protein causes disruption of intra-protein hydrophobic sites incurring the protein to fold into more helical state (Liu et al. 2004; Yamazaki et al. 2006; Lousa et al. 2012). Previously, we have demonstrated that alcohol inhibition to be among the reasons behind the low esterification activity of some lipases such as *Candida antarctica* lipase B (Gumel et al. 2011a, b). Thus, in this study, the observed decrease in esterification efficiency at higher methanol loading strongly indicated the same reason of alcohol inhibition. Similar inhibition of *C. antarctica* lipase B at higher methanol concentration was reported (Fjerbaek et al. 2009).

Alternatively, it could be possible that at higher acid concentrations (or lower alcohol:acid ratios), the hydrophobic interactions may cause the reduction in the catalytic turnover of the enzyme, hence lower esterification efficiency as reported previously (Lopresto et al. 2014). Furthermore, better *k*_cat_ value for methyl butanoate synthesis when compared to methyl benzoate synthesis may allow for more esterification to occur at higher concentration of butyric acid before the hydrophobic effects start to influence the reaction. For example in Fig. 5, during butanoate synthesis, it can be seen that the highest enzymatic esterification efficiency occurred at higher butanoic acid concentration (6.7 mM). Contrary to this, the highest esterification efficiency in benzoate synthesis occurred at lower benzoic acid concentration (4.8 mM).

Similar observations on the increase of enzymatic esterification efficiency with increasing substrate molar ratio were reported. For example, Liu et al. (2011a, b) reported 80 % increase in esterification efficiency by changing glycerol:oleic acid molar ratio to 7.5:1 during their study on lipase TL-catalyzed esterification of 1,3‐diacyl-glycerol in solvent‐free system. Furthermore, the alcohol:acid molar ratio (1.8) for highest esterification efficiency in our study was found to be in agreement with observation by Wang et al. (2011). The authors reported highest esterification efficiency of about 80 % at glycerol to free fatty acids molar ratio of 2 during diacylglycerol synthesis from aliphatic fatty acids by lipase TL-catalyzed esterification (Wang et al. 2011).

### Effect of enzyme loading on conversion yield

**Fig. 6:**
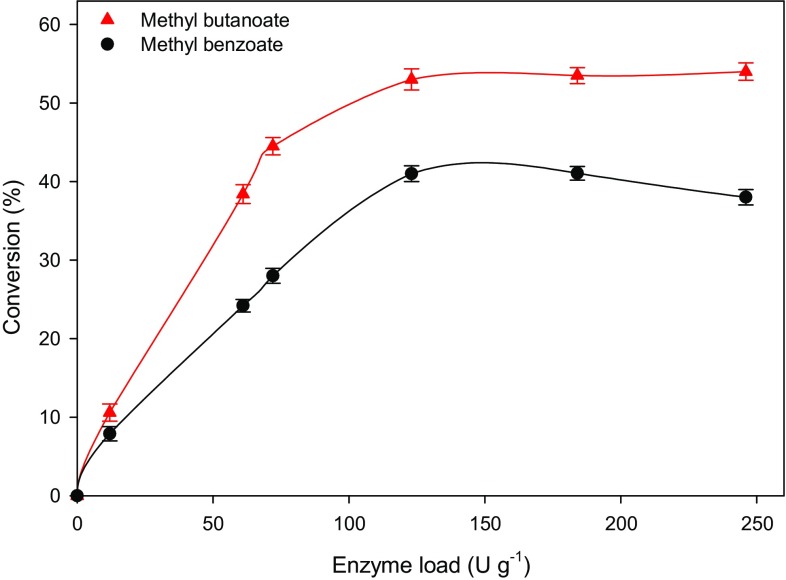
Effects of enzyme amount on conversion yield. Reaction conditions: acid (7 mM), methanol (12 mM), 0.1 mL min^−1^ flow rate, and 40 °C

The effects of enzyme loading on esterification of methyl esters of butanoate and benzoate in microfluidic reactor are shown in Fig. 6. Increase in enzyme loading to 123 U g^−1^ resulted in progressive increase in acid conversion achieving a maximum conversion of 54 and 41 % during the synthesis of methyl butanoate and methyl benzoate, respectively. Further increase in enzyme concentration beyond 123 U g^−1^ did not improve further the conversion yield for both esters (Fig. 6). This observation corroborated earlier reports (Liu et al. 2011a, b; Wang et al. 2011). For example, _ENREF_21_ENREF_13 Liu et al. (2011a, b) reported about 50 wt% increase in conversion by changing lipase TL loading from 0.5 to 1.5 wt%. Increasing the enzyme load beyond 1.5 wt% resulted in slight decrease of both percentage conversion and esterification efficiency. The possible reason for the reduction in conversion and esterification efficiency at higher lipase TL loading was explained by Wang et al. (2011). The researchers suggested that esterification reaction between immiscible alcohol and fatty acid is an interface reaction. At lower enzyme load, the alcohol/acid interface is unsaturated with enzyme molecules resulting in higher reaction rate, thus higher conversion. As the enzyme load increases, the interface is becoming more saturated with the adsorbed enzymes, resulting in diminished observable esterification rate (Wang et al. 2011).

### Influence of reaction temperature on reaction rate and conversion yield

**Fig. 7:**
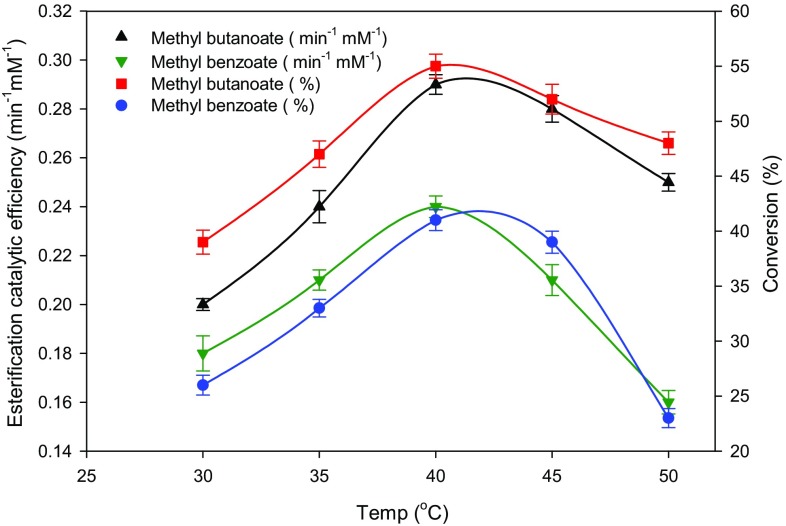
Effects of temperature as a function of esterification catalytic efficiency and conversion yield. Reaction conditions: acid (7 mM), methanol (12 mM), 0.1 mL min^−1^ flow rate, and 30 µL mL^−1^ lipase TL

In biocatalysis, temperature has great influence on the catalytic activity of the enzyme and the thermodynamic equilibrium of a reaction. The effect of reaction temperature ranging from 30 to 50 °C on lipase TL-catalyzed esterification of flavor esters in microfluidic process is shown in Fig. 7. When the reaction temperature was increased from 30 to 40 °C, steady increase in esterification catalytic efficiency and conversion yield was observed. Higher *E*_cat_ (0.3 min^−1^ mM^−1^) and conversion (54 %) were observed in methyl butanoate synthesis compared to the synthesis of methyl benzoate (0.24 min^−1^ mM^−1^ and 41 %). When the temperature was increased beyond 40 °C, marked decrease in the esterification efficiency and conversion yield was observed in both ester syntheses (Fig. 7). This observation was found to be in accordance with the previous literatures (Yang et al. 2006; Liu et al. 2011a, b; Wang et al. 2011) reporting a temperature limit of 40 °C for lipase TL catalysis. Beyond 40 °C, they observed a significant decline in the reaction efficiency and yield. The observed decrease in efficiency and conversion yield at temperatures beyond 40 °C could be due to the following: (1) reported increase in phospholipase activity over the lipase activity of lipase TL when temperature is higher than 40 °C (Yang et al. 2006) or (2) due to thermal deactivation effects (Mishra et al. 2009).

## Conclusions

The application of lipase TL in the esterification synthesis of methyl butanoate and methyl benzoate in a microfluidic process was demonstrated as a model system for the synthesis of other valuable esters. The following reaction conditions were 40 °C, flow rate 0.1 mL min^−1^, and 123 U g^−1^ enzyme loading found to be the optimum operating limits. The work demonstrated the application of enzyme(s) in a microreactor system for the synthesis of industrially important esters. While there is room for improvement in terms of conversion yield, the major advantage is the absence of side reaction(s) and by-product(s) owing to the specificity of the enzyme-catalyzed process.

## Abbreviations

*k*_cat_/*K*_M_: Catalytic efficiency (min^−1^ mM^−1^)
*k*_cat_: Catalytic turnover (min^−1^)
*v*: Initial reaction rate (mM min^−1^)
*K*_M_: Michaelis constant (mM)
*V*_max_: Maximum reaction rate (mM min^−1^)
*Pe*: Péclet number (dimensionless)
*Da*: Damköhler numbers (dimensionless)
*Re*: Reynolds number (dimensionless)
*D*: Diffusivity coefficient (cm^2^ s^−1^)
*D*_eff_: Effective diffusivity (cm^2^ s^−1^)
*U*: Superficial Velocity (cm s^−1^)
*q*_v_: Volumetric flow rate (cm^3^ min^−1^)
$\bar{V}_{\text{S}}$: Molar volume of solvent (cm^3^ mol^−1^)
$\bar{V}_{\text{r}}$: Molar volume of reactant (cm^3^ mol^−1^)
$\eta_{\text{S}}$: Dynamic viscosity of solvent (mPa s)
*t*_r_: Characteristic reaction time (s)
*l*: Channel characteristic length (cm)
*r*: Radius of the channel (cm)
*P*: Circumference of the channel (cm)
